# 81941 Evaluating race, socioeconomic status, and the effect of radiation treatment in patients undergoing autologous breast reconstruction

**DOI:** 10.1017/cts.2021.748

**Published:** 2021-03-30

**Authors:** Edgar Soto, Grant Bond, Jeremy Botwsorth, Hua A. Fang, Rene P. Myers, Timothy W. King

**Affiliations:** University of Alabama School of Medicine

## Abstract

ABSTRACT IMPACT: Disparities are multifactorial in etiology we seek to elucidate the effects of social determinants of health such as race on the outcomes of autologous flap reconstruction. OBJECTIVES/GOALS: Immediate breast reconstruction has increased in recent years yet, racial and socioeconomic disparities in the receipt of postmastectomy breast reconstruction persist. We review the usage of autologous flaps for immediate breast reconstruction in a single institution with a diverse population to determine the effect of radiation on flap survival. METHODS/STUDY POPULATION: The database of a Southeastern tertiary referral center was queried for patients who received autologus flaps for immediate reconstruction following mastectomy. Patients were stratified based on whether they received no radiation (TRAM), neoadjuvant radiation (TRAM + Pre-XRT), or post-reconstruction radiation (TRAM + PMRT). So far, we have identified 91 patients (157 breasts) meeting inclusion criteria from 2006 to 2017. Patient demographics and outcomes were compared based on radiation status. The primary outcome (reconstructive success) was defined as breast reconstruction without flap loss. Comorbidities, socioeconomic status, and method of reconstruction were collected. Statistical analysis included t-tests, chi-square tests and logistic regression were appropriate using R. RESULTS/ANTICIPATED RESULTS: At the moment, we focus on outcomes of transverse rectus abdominus flaps and are adding information on 4 other flap-based methods. There were 68 in the solely TRAM group, 33 in TRAM+Pre-XRT and 56 in TRAM+PMRT with equivalent demographics between all groups for Age, Race and BMI (Table 1). In terms of race most patients self-identified as White (68%), followed by Black (24%) and Other (8%), p=0.172. There was a statistically significant difference in the incidence of tobacco use with the type of radiation used (p=0.007) with the PTRAM+ PMRT group having the highest percentage. When analyzing major and minor complications based on radiation received or reconstructive success there was no significant difference regardless of radiation treatment with the group overall achieving a 97.4% success rate (p=0.229). DISCUSSION/SIGNIFICANCE OF FINDINGS: Despite the known racial disparities in healthcare and the deleterious effects of radiation therapy on wound healing, there was no significant difference found in the incidence of major or minor complications in patients receiving neoadjuvant or post-reconstruction radiation therapy regardless of patient demographics.

